# Permanent draft genome sequences of the symbiotic nitrogen fixing *Ensifer meliloti* strains BO21CC and AK58

**DOI:** 10.4056/sigs.3797438

**Published:** 2013-12-15

**Authors:** Marco Galardini, Marco Bazzicalupo, Emanuele Biondi, Eveline Brambilla, Matteo Brilli, David Bruce, Patrick Chain, Amy Chen, Hajnalka Daligault, Karen Walston Davenport, Shweta Deshpande, John C. Detter, Lynne A. Goodwin, Cliff Han, James Han, Marcel Huntemann, Natalia Ivanova, Hans-Peter Klenk, Nikos C. Kyrpides, Victor Markowitz, Kostas Mavrommatis, Stefano Mocali, Matt Nolan, Ioanna Pagani, Amrita Pati, Francesco Pini, Sam Pitluck, Giulia Spini, Ernest Szeto, Hazuki Teshima, Tanja Woyke, Alessio Mengoni

**Affiliations:** 1 Department of Biology, University of Firenze, via Madonna del Piano 6, I-50019, Sesto Fiorentino, Italy; 2 Interdisciplinary Research Institute - CNRS, Villenenuve d'Ascq, France; 3 Leibniz Institute DSMZ - German Collection of Microorganisms and Cell Cultures, Braunschweig, Germany; 4 Edmund Mach Foundation, San Michele all’Adige, Italy; 5 Los Alamos National Laboratory, Bioscience Division, Los Alamos, New Mexico, USA; 6 DOE Joint Genome Institute, Walnut Creek, California, USA; 7 Consiglio per la Ricerca e la Sperimentazione in Agricoltura - Centro di Ricerca per l’Agropedologia e la Pedologia, Firenze, Italy

**Keywords:** Aerobic, motile, Gram-negative, mesophilic, chemoorganotrophic, chemoautotrophic, soil, plant symbiont, biological nitrogen fixation, *Ensifer* (*Sinorhizobium) meliloti*, legume yield

## Abstract

*Ensifer* (syn. *Sinorhizobium*) *meliloti* is an important symbiotic bacterial species that fixes nitrogen. Strains BO21CC and AK58 were previously investigated for their substrate utilization and their plant-growth promoting abilities showing interesting features. Here, we describe the complete genome sequence and annotation of these strains. BO21CC and AK58 genomes are 6,985,065 and 6,974,333 bp long with 6,746 and 6,992 genes predicted, respectively.

## Introduction

Strains AK58 and BO21CC belong to the species *Ensifer* (syn. *Sinorhizobium*) *meliloti* (*Alphaproteobacteria*, *Rhizobiales*, *Rhizobiaceae*, *Sinorhizobium*/*Ensifer* group) [1,2], an important symbiotic nitrogen fixing bacterial species that associates with roots of leguminous plants of several genera, mainly from *Melilotus*, *Medicago* and *Trigonella* [3]. These strains have been originally isolated from *Medicago* spp. during a long course experiment (BO21CC) and from plants collected in the north Aral sea region (Kazakhstan) (AK58). Previous analyses conducted by comparative genomic hybridization (CGH), nodulation tests and Phenotype Microarray™(Biolog, Inc.) showed that AK58 (= DSM 23808) and BO21CC (= DSM 23809) are highly diverse in both genomic and phenotypic properties. In particular, they show different symbiotic phenotypes with respect to the crop legume *Medicago sativa* L [4,5]. In a previous collaboration with DOE-JGI, the genomes of strains AK83 (= DSM 23913) and BL225C (= DSM 23914) were also sequenced, allowing the identification of putative genetic determinants for their different symbiotic phenotypes [6]. Consequently, interest in strains AK58 and BO21CC arose, since genomic analysis of these strains would foster a greater understanding of the *E. meliloti* pangenome [7], and facilitate deeper investigation of the genomic determinants responsible for differences in symbiotic performances between *E. meliloti* strains found in nature. These research goals may lead to improved strain selection and better inoculants of the legume crop *M. sativa*.

## Classification and features

**Figure 1:**
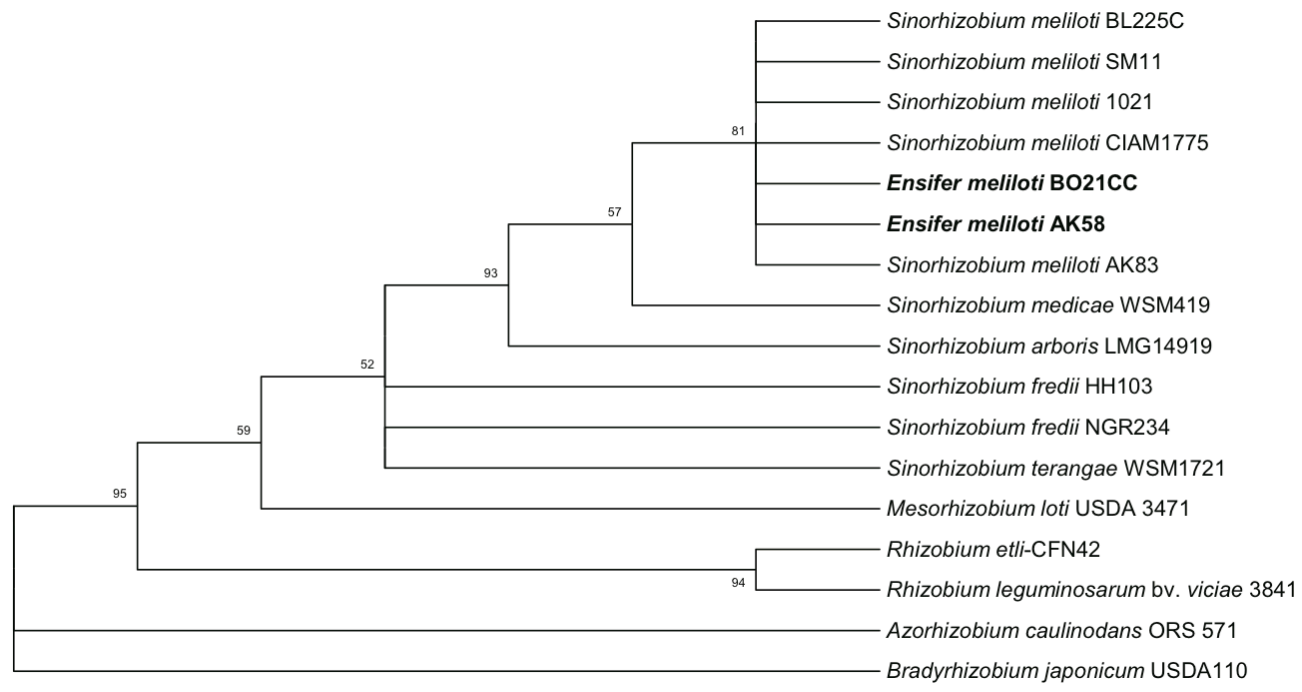
Phylogenetic consensus tree showing the position of *E. meliloti* AK58 and BO21CC strains in the *Ensifer*/*Sinorhizobium* genus. The phylogenetic tree was inferred by using the Maximum Likelihood method based on the Tamura 3-parameter model [17], chosen as model with the lowest BIC scores (Bayesian Information Criterion) after running a Maximum Likelihood fits of 24 different nucleotide substitution models (Model Test). The bootstrap consensus tree inferred from 500 replicates [18] is taken to represent the phylogenetic pattern of the taxa analyzed [18]. Branches corresponding to partitions reproduced in less than 50% bootstrap replicates are collapsed. The percentage of replicate trees in which the associated taxa clustered together in the bootstrap test (500 replicates) are shown next to the branches. The tree with the highest log likelihood (-3411.7124) is shown. The percentage of trees in which the associated taxa clustered together is shown next to the branches. A discrete Gamma distribution was used to model evolutionary rate differences among sites (G, parameter = 0.3439). A total of 1,284 nt positions were present in the final dataset. Model test and Maximum Likelihood inference were conducted in MEGA5 [19]. In bold *E. meliloti* AK58 and BO21CC strains.

Representative genomic 16S rRNA sequences of strains AK58 and BO21CC were compared with those present in the Ribosomal Database by using Match Sequence module of Ribosomal Database Project [8]. Representative genomic 16S rRNA sequences of closer phylogenetic relatives of the genus *Ensifer*/*Sinorhizobium* and of *Rhizobiales* family (as outgroup) were then selected from IMG-ER database [Table 1] [16]. All strains from the genus *Ensifer*/*Sinorhizobium* form a close cluster, including strains AK58 and BO21CC, thus confirming the affiliation of these two strains within the species. Figure 1 shows the phylogenetic neighborhood of *E. meliloti* AK58 and BO21CC in a 16S rRNA based tree.

**Table 1:** Classification and general features of *E. meliloti* AK58 and BO21CC according to the MIGS recommendations [9] and the Names for Life database [10]

**MIGS ID**	**Property**	**Term**	**Evidence code**
	Current classification	Domain *Bacteria*	TAS [11]
		Phylum *Proteobacteria*	TAS [12]
		Class *Alphaproteobacteria*	TAS [12]
		Order *Rhizobiales*	TAS [12]
		Family *Rhizobiaceae*	TAS [12]
		Genus *Ensifer*	TAS [2,12]
		Species *Ensifer meliloti*	TAS [13]
		Strain BO21CC Strain AK58	TAS [4,5]
	Gram stain	negative	TAS [12]
	Cell shape	rods	TAS [12]
	Motility	Motile	TAS [12]
	Sporulation	non-sporulating	TAS [12]
	Temperature range	mesophile, 20-37°C	TAS [12]
	Optimum temperature	25-30°C	TAS [12]
	Salinity	Tolerate 1.0% NaCl	TAS [12]
MIGS-22	Oxygen requirement	Aerobe	TAS [12]
	Carbon source	carbohydrates and salts of organic acids	TAS [12]
	Energy metabolism	chemoorganotroph	TAS [12]
MIGS-6	Habitat	Soil, root nodules of legumes	TAS [3,12]
MIGS-15	Biotic relationship	free living, symbiont	TAS [12]
MIGS-14	Pathogenicity	not reported	
	Biosafety level	1	TAS [14]
MIGS-23.1	Isolation	BO21CC: root nodules of *Medicago sativa* cv. ‘Oneida’ AK58: root nodules of *Medicago falcata*	TAS [4]
MIGS-4	Geographic location	BO21CC: Lodi, Italy AK58: Kazakhstan,	TAS [4]
MIGS-5	Sample collection time	BO21CC: 1997 AK58: 2001	NAS
MIGS-4.1	Latitude	BO21CC: 45.31 AK58: 58.75	NAS
MIGS-4.2	Longitude	BO21CC: 9.50 AK58: 48.98	NAS
MIGS-4.3	Depth	not reported	
MIGS-4.4	Altitude	BO21CC: 70 m AK58: 305 m	NAS

Evidence codes - TAS: Traceable Author Statement (i.e., a direct report exists in the literature); NAS: Non-traceable Author Statement (i.e., not directly observed for the living, isolated sample, but based on a generally accepted property for the species, or anecdotal evidence). These evidence codes are from the Gene Ontology project [15].

**Figure 2:**
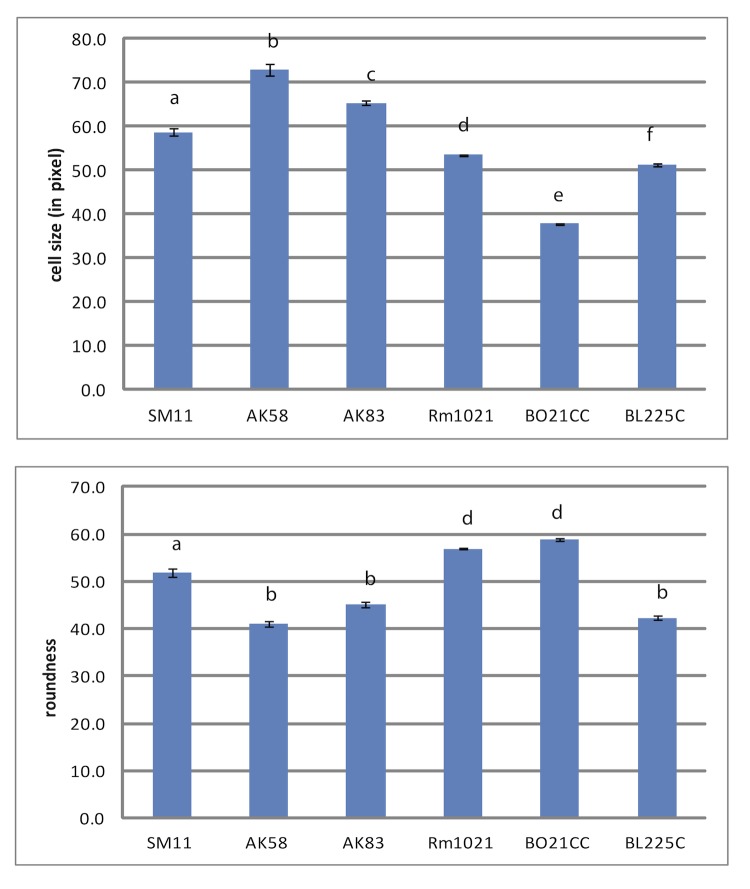
Cell morphology and cell size analysis of *E. meliloti* strains. Cell size analysis with Pixcavator IA 5.1.0.0 software [20] of logarithmically grown cultures (OD_600_=0.6) in TY medium of AK58, BO21CC, plus other completely sequenced *E. meliloti* strains is reported. Cell size is expressed as cell area in µm^2^, while roundness is the ratio between the two main axes of the cell. Standard errors after more than 300 individual observations are reported. Different letters indicate significant differences (P<0.05) after 1-way ANOVA.

*E. meliloti* AK58 and BO21CC show different symbiotic phenotypes with respect to the host plant *Medicago sativa*, as well as differences in substrates utilization [5]. Moreover *E. meliloti* AK58 and BO21CC present differences in cell morphology also, with AK58 being smaller than BO21CC and the other *E. meliloti* strains for which genome sequencing is available (Figure 2). Interestingly, BO21CC is also showing cells with a ratio between cell axes nearer 1 (more rounded cells), when compared with AK58 and with the other *E. meliloti* strains (Figure 2).

## Genome sequencing information

### Genome project history

AK58 and BO21CC strains were selected for sequencing on the basis of the Community Sequencing Program 2010 of DOE Joint Genome Institute (JGI) in relation to the project entitled “Complete genome sequencing of *Sinorhizobium meliloti* AK58 and BO21CC strains: Improving alfalfa performances through the exploitation of *Sinorhizobium* genomic data”. The overall rationale for their genome sequencing was related to the identification of genomic determinants of different symbiotic performances between *S. meliloti* strains. The genome project is deposited in the Genomes On Line Database [21] and the complete genome sequence is deposited in GenBank. Sequencing, finishing and annotation were performed by the DOE-JGI. A summary of the project information is shown in Table 2.

**Table 2:** Genome sequencing project information

**MIGS ID**	**Property**	**Term**
MIGS-31	Finishing quality	High-Quality Draft
MIGS-28	Libraries used	Two genomic libraries: one 454 PE library (9 kb insert size), one Illumina library
MIGS-29	Sequencing platforms	Illumina GAii, 454 GS FLX Titanium
MIGS-31.2	Sequencing coverage	60 × (AK58) 910 × (BO21CC) Illumina; 8.8 × pyrosequence
MIGS-30	Assemblers	Newbler version 2.3, Velvet version 1.0.13, phrap version, 1.080812, Allpaths version 39750,
MIGS-32	Gene calling method	Prodigal
	GenBank Date of Release	BO21CC: AXBL01000000 AK58: AXBM00000000
	GOLD ID	BO21CC: Gi07569 AK58: Gi07577
	NCBI project ID	BO21CC: 375171 AK58: 928722
	Database: IMG	BO21CC: 9144 AK58: 7327
MIGS-13	Source material identifier	BO21CC: DSM23809 AK58: DSM23808
	Project relevance	CSP2010, biotechnological, biodiversity

### Growth conditions and DNA isolation

*E. meliloti* strains AK58 and BO21CC (DSM23808 and DSM23809, respectively) were grown in DSMZ medium 98 (Rhizobium medium) [22] at 28°C. DNA was isolated from 0.5-1 g of cell paste using Jetflex Genomic DNA Purification kit (GENOMED 600100) following the standard protocol as recommended by the manufacturer with modification st/LALMP [23] for strain AK58 and additional 5 µl proteinase K incubation at 58° for 1 hour for strain BO21CC, respectively. DNA will be available on request through the DNA Bank Network [24].

### Genome sequencing and assembly

The draft genomes were generated at the DOE Joint Genome Institute (JGI) using Illumina data [25]. For BO21CC genome, we constructed and sequenced an Illumina short-insert paired-end library with an average insert size of 270 bp which generated 76,033,356 reads and an Illumina long-insert paired-end library with an average insert size of 9,141.74 ± 1,934.63 bp, which generated 4,563,348 reads totaling 6,463 Mbp of Illumina data. For AK58, a combination of Illumina [25] and 454 technologies [26] was used. For the AK58 genome we constructed and sequenced an Illumina GAii shotgun library which generated 80,296,956 reads totaling 6,102.6 Mb, a 454 Titanium standard library which generated 0 reads and 1 paired end 454 library with an average insert size of 10 kb, which generated 326,569 reads totaling 96 Mb of 454 data. All general aspects of library construction and sequencing performed at the JGI can be found at the JGI website [27]. The initial draft assemblies contained 194 contigs in 16 scaffold(s) for BO21CC, and 311 contigs in 5 scaffolds for AK58.

For BO21CC the initial draft data was assembled with Allpaths and the consensus was computationally shredded into 10 Kbp overlapping fake reads (shreds). The Illumina draft data was also assembled with Velvet, version 1.1.05 [28], and the consensus sequences were computationally shredded into 1.5 Kbp overlapping fake reads (shreds). The Illumina draft data was assembled again with Velvet using the shreds from the first Velvet assembly to guide the next assembly. The consensus from the second Velvet assembly was shredded into 1.5 Kbp overlapping fake reads. The fake reads from the Allpaths assembly and both Velvet assemblies and a subset of the Illumina CLIP paired-end reads were assembled using parallel phrap, version 4.24 (High Performance Software, LLC). Possible mis-assemblies were corrected with manual editing in Consed [29-31]. 

Gap closure was accomplished using repeat resolution software (Wei Gu, unpublished), and sequencing of bridging PCR fragments with Sanger and/or PacBio (unpublished, Cliff Han) technologies. For improved high quality draft and noncontiguous finished projects, one round of manual/wet lab finishing may have been completed. Primer walks, shatter libraries, and/or subsequent PCR reads may also be included for a finished project. A total of 128 additional sequencing reactions and 126 PCR PacBio consensus sequences were completed to close gaps and to raise the quality of the final sequence. The total ("estimated size" for unfinished) size of the BO21CC genome is 7.1 Mb and the final assembly is based on 6,463 Mbp of Illumina draft data, which provides an average 910 × coverage of the genome.

For AK58, the 454 Titanium standard data and the 454 paired end data were assembled together with Newbler, version 2.6 (20110517_1502). The Newbler consensus sequences were computationally shredded into 2 kb overlapping fake reads (shreds). Illumina sequencing data was assembled with Velvet, version 1.1.05 [28], and the consensus sequence was computationally shredded into 1.5 kb overlapping fake reads (shreds). We integrated the 454 Newbler consensus shreds, the Illumina Velvet consensus shreds and the read pairs in the 454 paired end library using parallel phrap, version SPS - 4.24 (High Performance Software, LLC). The software Consed [29-31] was used in the following finishing process. Illumina data was used to correct potential base errors and increase consensus quality using the software Polisher developed at JGI (Alla Lapidus, unpublished). Possible mis-assemblies were corrected using gapResolution (Cliff Han, unpublished), Dupfinisher [32], or sequencing cloned bridging PCR fragments with subcloning. Gaps between contigs were closed by editing in Consed, by PCR and by Bubble PCR (J-F Cheng, unpublished) primer walks. A total of 0 additional reactions were necessary to close gaps and to raise the quality of the finished sequence. The estimated genome size of AK58 is 7 Mb and the final assembly is based on 61.5 Mb of 454 draft data which provides an average 8.8 × coverage of the genome and 420 Mb of Illumina draft data which provides an average 60 × coverage of the genome.

### Genome annotation

Genes were identified using Prodigal [33] as part of the Oak Ridge National Laboratory genome annotation pipeline, followed by a round of manual curation using the JGI GenePRIMP pipeline [34]. The predicted CDSs were translated and used to search the National Center for Biotechnology Information (NCBI) non-redundant database, UniProt, TIGRFam, Pfam, PRIAM, KEGG, COG, and InterPro databases. Additional gene prediction analysis and functional annotation was performed within the Integrated Microbial Genomes - Expert Review (IMG-ER) platform [16].

## Genome properties

The High-Quality draft assemblies of the genomes consist of 41 scaffolds for BO21CC and 9 scaffolds for AK58 representing overall 6,985,065 and 6,974,333 bp, respectively. The overall G+C content was 62.12% and 62.04% for BO21CC and AK58, respectively (Table 3a and Table 3b). Of the 6,746 and 6,992 genes predicted, 5,357 and 5,549 were protein-coding genes, and 105 and 79 RNAs were present in BO21CC and AK58, respectively. The large majority of the protein-coding genes (79.32% and 78.03%, BO21CC and AK58, respectively) were assigned a putative function as COGs. The distribution of genes into COGs functional categories is presented in Table 4.

**Table 3a:** Genome Statistics for strain BO21CC

**Attribute**	**Value**	**% of Total**
Genome size (bp)	6,985,065	100.00%
DNA coding region (bp)	6,011,953	86.07%
DNA G+C content (bp)	4,339,356	62.12%
Number of scaffolds	41	
Total genes	6,746	100.00%
RNA genes	105	1.72%
rRNA operons	3	
tRNA genes	58	0.86%
Protein-coding genes		
Genes with function prediction (proteins)	5,357	79.41%
Genes in paralog clusters	3,275	48.55%
Genes assigned to COGs	5,351	79.32%
Genes assigned Pfam domains	5,318	78.83%
Genes with signal peptides	1,427	21.15%
Genes with transmembrane helices	1,521	22.55%

**Table 3b:** Genome statistics for strain AK58

**Attribute**	**Value**	**%age**
Genome size (bp)	6,974,333	100.00%
DNA coding region (bp)	5,914,246	84.80%
DNA G+C content (bp)	4,315,694	62.04%
Number of scaffolds	9	
Total genes	6,992	100.00%
RNA genes	79	1.13%
rRNA operons	1*	
tRNA genes	49	0.70%
Protein-coding genes	6,934	98.87%
Genes with function prediction (proteins)	5,459	77.84%
Genes in paralog clusters	2,912	41.52%
Genes assigned to COGs	5,472	78.03%
Genes assigned Pfam domains	5,420	77.29%
Genes with signal peptides	1,432	20.42% %
Genes with transmembrane helices	1,465	20.89%

*only one rRNA operon appears to be complete.

**Table 4:** Number of genes associated with the general COG functional categories

	BO21CC		AK58		
**Code**	**Value**	**%age**	**Value**	**%age**	**Description**
J	195	3.27	201	3.29	Translation, ribosomal structure and biogenesis
A	-	-	-	-	RNA processing and modification
K	524	8.79	551	9.01	Transcription
L	273	4.58	327	5.35	Replication, recombination and repair
B	1	0.02	3	0.05	Chromatin structure and dynamics
D	45	0.76	53	0.87	Cell cycle control, cell division, chromosome partitioning
Y	-	-	-	-	Nuclear structure
V	64	1.17	62	1.01	Defense mechanisms
T	247	4.14	249	4.07	Signal transduction mechanisms
M	305	5.12	298	4.87	Cell wall/membrane biogenesis
N	69	1.16	68	1.11	Cell motility
Z	-	-	-	-	Cytoskeleton
W	1	0.02	1	0.02	Extracellular structures
U	104	1.75	102	1.67	Intracellular trafficking and secretion, and vesicular transport
O	185	3.10	189	3.09	Posttranslational modification, protein turnover, chaperones
C	365	6.13	356	5.82	Energy production and conversion
G	604	10.14	596	9.75	Carbohydrate transport and metabolism
E	637	10.69	685	11.20	Amino acid transport and metabolism
F	107	1.80	114	1.86	Nucleotide transport and metabolism
H	202	3.39	205	3.35	Coenzyme transport and metabolism
I	210	3.52	217	3.55	Lipid transport and metabolism
P	320	5.17	294	4.81	Inorganic ion transport and metabolism
Q	163	2.74	159	2.60	Secondary metabolites biosynthesis, transport and catabolism
R	730	12.25	767	12.54	General function prediction only
S	608	10.20	617	10.09	Function unknown
					
					
